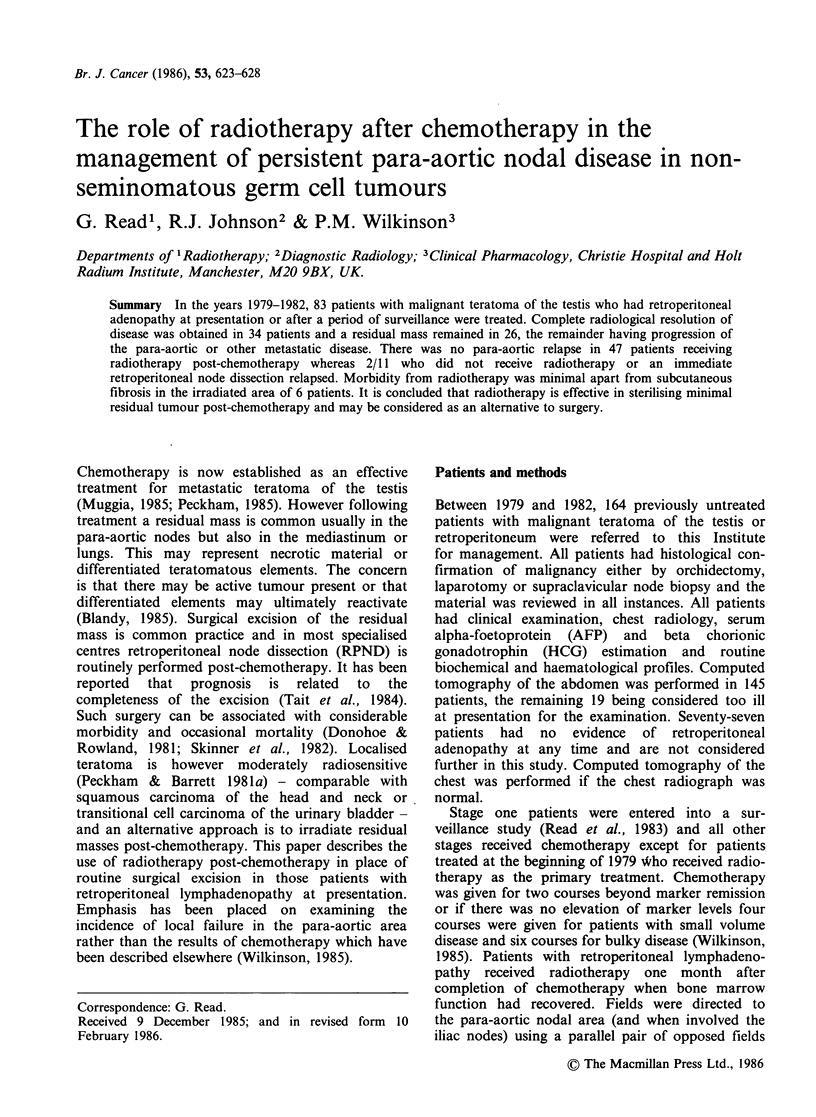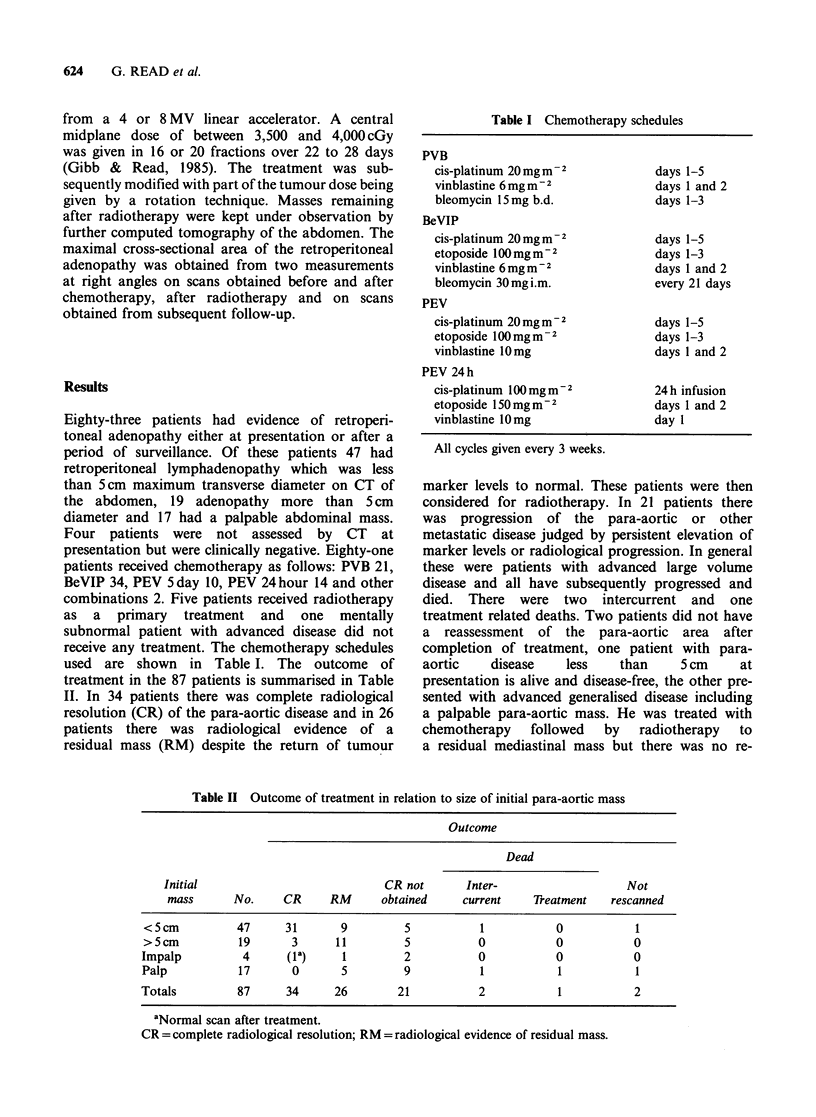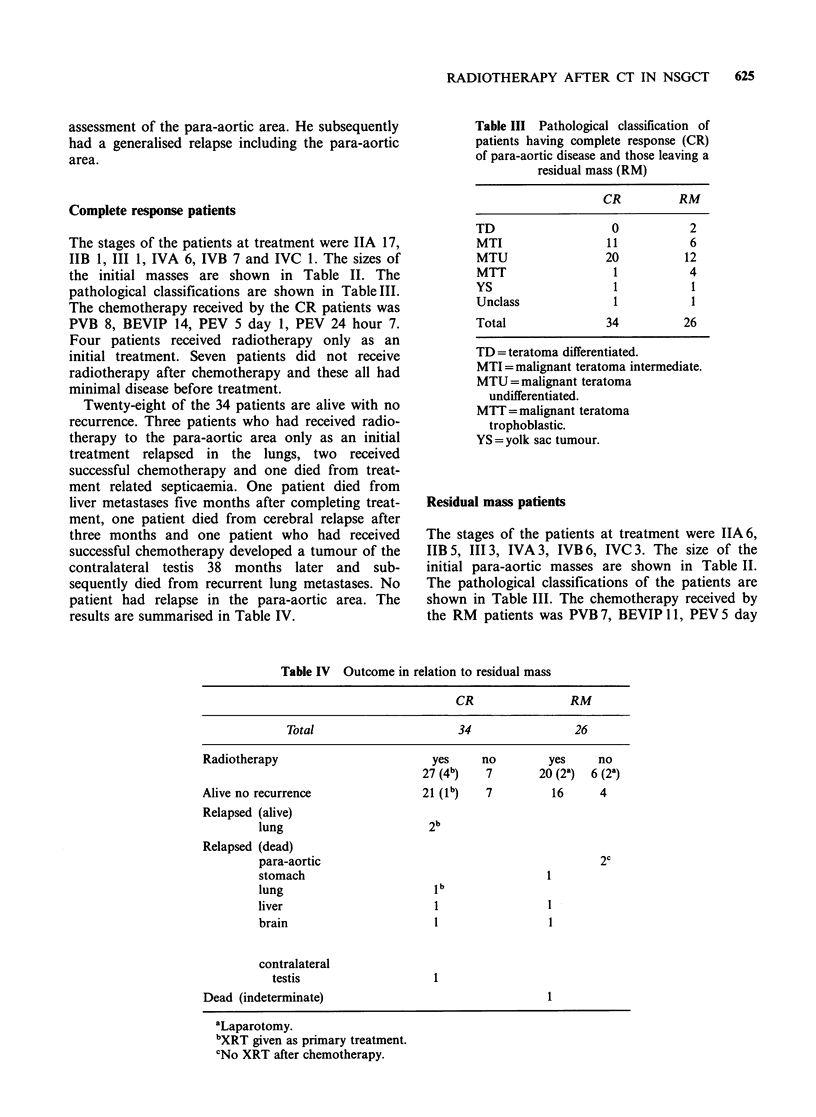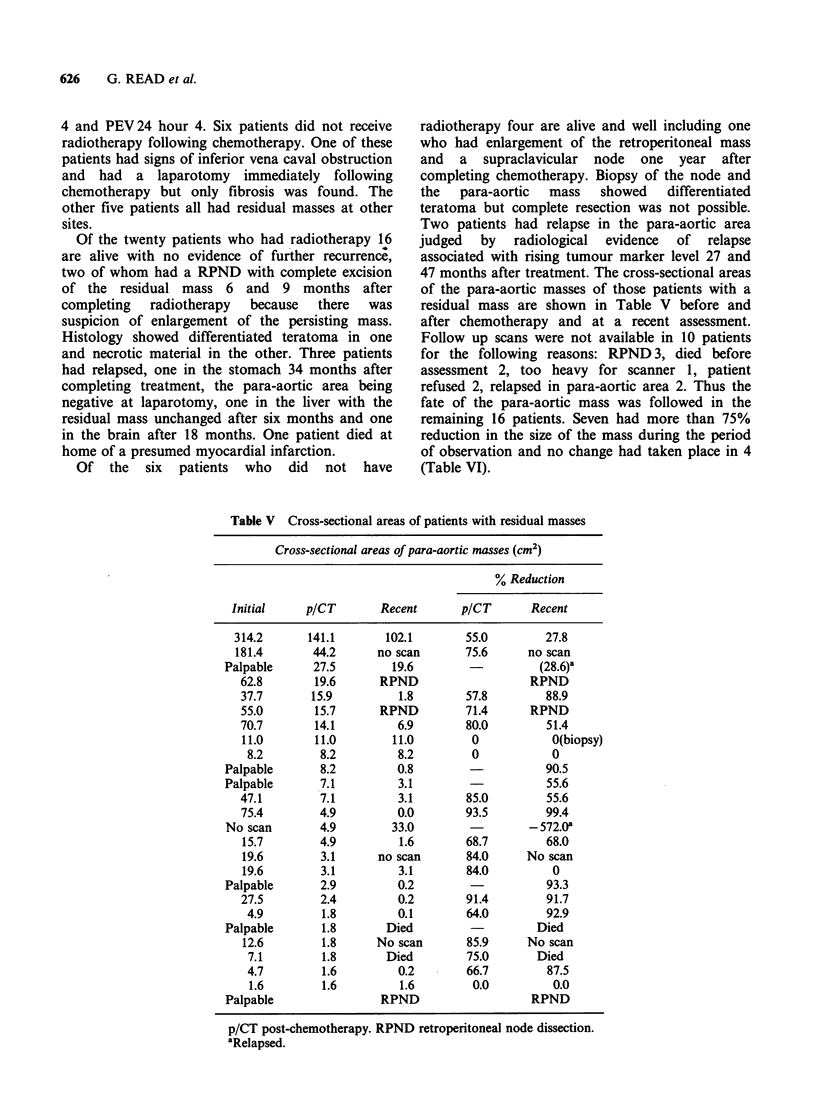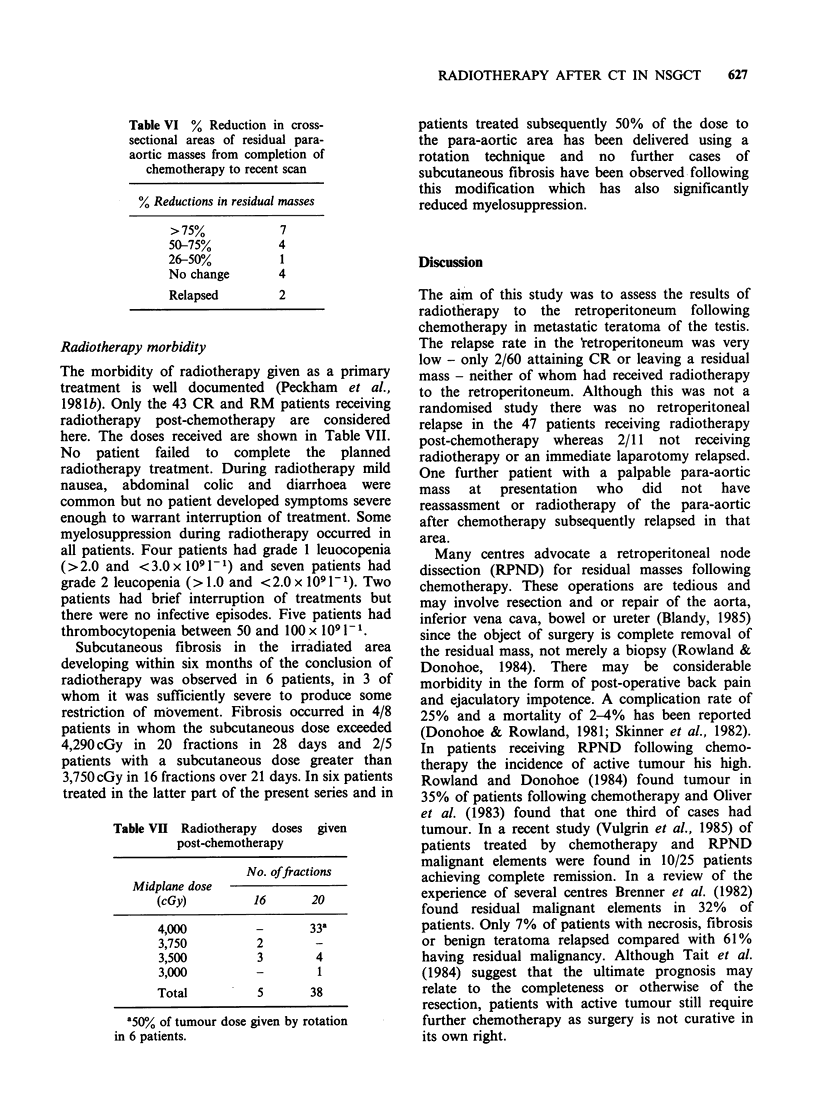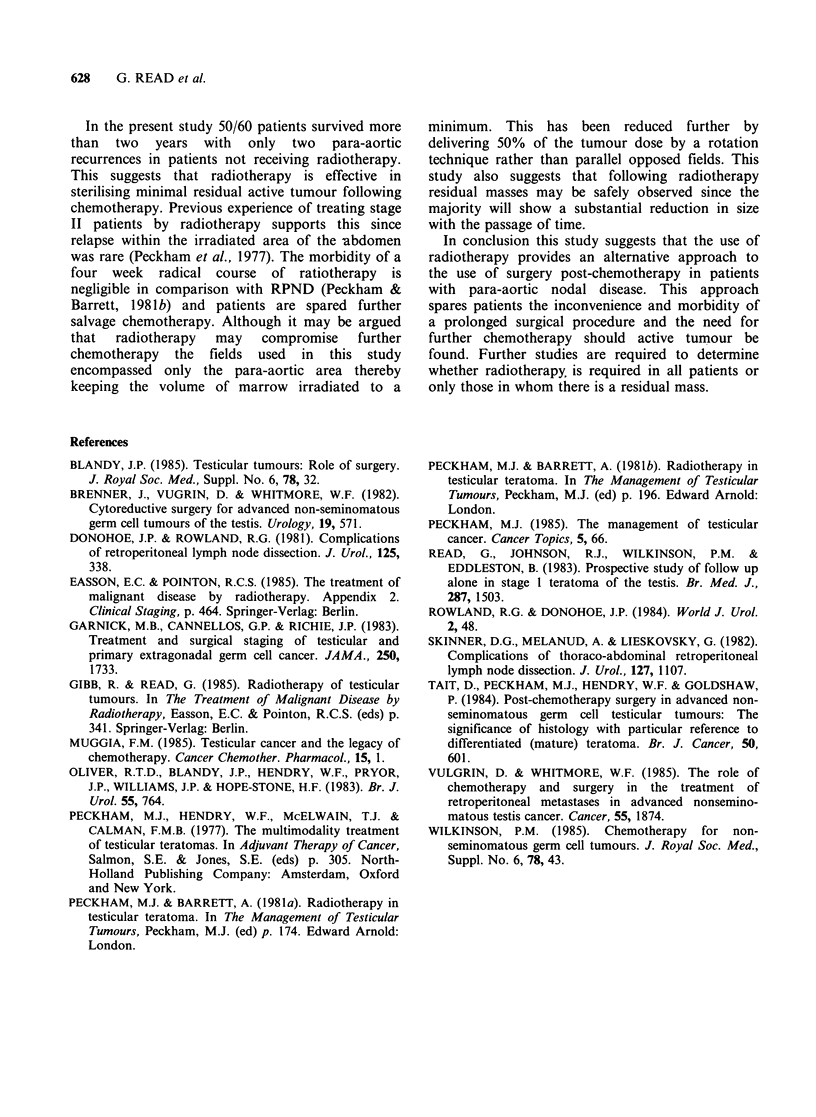# The role of radiotherapy after chemotherapy in the management of persistent para-aortic nodal disease in non-seminomatous germ cell tumours.

**DOI:** 10.1038/bjc.1986.105

**Published:** 1986-05

**Authors:** G. Read, R. J. Johnson, P. M. Wilkinson

## Abstract

In the years 1979-1982, 83 patients with malignant teratoma of the testis who had retroperitoneal adenopathy at presentation or after a period of surveillance were treated. Complete radiological resolution of disease was obtained in 34 patients and a residual mass remained in 26, the remainder having progression of the para-aortic or other metastatic disease. There was no para-aortic relapse in 47 patients receiving radiotherapy post-chemotherapy whereas 2/11 who did not receive radiotherapy or an immediate retroperitoneal node dissection relapsed. Morbidity from radiotherapy was minimal apart from subcutaneous fibrosis in the irradiated area of 6 patients. It is concluded that radiotherapy is effective in sterilising minimal residual tumour post-chemotherapy and may be considered as an alternative to surgery.


*Br. J. Cancer* (1986), **53**, 623–628

### The role of radiotherapy after chemotherapy in the management of persistent para-aortic nodal disease in non-seminomatous germ cell tumours

G. Read[1], R.J. Johnson[2] & P.M. Wilkinson[3]

*Departments of [1]Radiotherapy; [2]Diagnostic Radiology; [3]Clinical Pharmacology, Christie Hospital and Holt Radium Institute, Manchester, M20 9BX, UK.*

**Summary** In the years 1979–1982, 83 patients with malignant teratoma of the testis who had retroperitoneal adenopathy at presentation or after a period of surveillance were treated. Complete radiological resolution of disease was obtained in 34 patients and a residual mass remained in 26, the remainder having progression of the para-aortic or other metastatic disease. There was no para-aortic relapse in 47 patients receiving radiotherapy post-chemotherapy whereas 2/11 who did not receive radiotherapy or an immediate retroperitoneal node dissection relapsed. Morbidity from radiotherapy was minimal apart from subcutaneous fibrosis in the irradiated area of 6 patients. It is concluded that radiotherapy is effective in sterilising minimal residual tumour post-chemotherapy and may be considered as an alternative to surgery.

Chemotherapy is now established as an effective treatment for metastatic teratoma of the testis (Muggia, 1985; Peckham, 1985). However following treatment a residual mass is common usually in the para-aortic nodes but also in the mediastinum or lungs. This may represent necrotic material or differentiated teratomatous elements. The concern is that there may be active tumour present or that differentiated elements may ultimately reactivate (Blandy, 1985). Surgical excision of the residual mass is common practice and in most specialised centres retroperitoneal node dissection (RPND) is routinely performed post-chemotherapy. It has been reported that prognosis is related to the completeness of the excision (Tait *et al.*, 1984). Such surgery can be associated with considerable morbidity and occasional mortality (Donohoe & Rowland, 1981; Skinner *et al.*, 1982). Localised teratoma is however moderately radiosensitive (Peckham & Barrett 1981*a*) – comparable with squamous carcinoma of the head and neck or transitional cell carcinoma of the urinary bladder – and an alternative approach is to irradiate residual masses post-chemotherapy. This paper describes the use of radiotherapy post-chemotherapy in place of routine surgical excision in those patients with retroperitoneal lymphadenopathy at presentation. Emphasis has been placed on examining the incidence of local failure in the para-aortic area rather than the results of chemotherapy which have been described elsewhere (Wilkinson, 1985).

Correspondence: G. Read.
Received 9 December 1985; and in revised form 10 February 1986.

### Patients and methods

Between 1979 and 1982, 164 previously untreated patients with malignant teratoma of the testis or retroperitoneum were referred to this Institute for management. All patients had histological confirmation of malignancy either by orchidectomy, laparotomy or supraclavicular node biopsy and the material was reviewed in all instances. All patients had clinical examination, chest radiology, serum alpha-foetoprotein (AFP) and beta chorionic gonadotrophin (HCG) estimation and routine biochemical and haematological profiles. Computed tomography of the abdomen was performed in 145 patients, the remaining 19 being considered too ill at presentation for the examination. Seventy-seven patients had no evidence of retroperitoneal adenopathy at any time and are not considered further in this study. Computed tomography of the chest was performed if the chest radiograph was normal.

Stage one patients were entered into a surveillance study (Read *et al.*, 1983) and all other stages received chemotherapy except for patients treated at the beginning of 1979 who received radiotherapy as the primary treatment. Chemotherapy was given for two courses beyond marker remission or if there was no elevation of marker levels four courses were given for patients with small volume disease and six courses for bulky disease (Wilkinson, 1985). Patients with retroperitoneal lymphadenopathy received radiotherapy one month after completion of chemotherapy when bone marrow function had recovered. Fields were directed to the para-aortic nodal area (and when involved the iliac nodes) using a parallel pair of opposed fields

© The Macmillan Press Ltd., 1986

from a 4 or 8 MV linear accelerator. A central midplane dose of between 3,500 and 4,000 cGy was given in 16 or 20 fractions over 22 to 28 days (Gibb & Read, 1985). The treatment was subsequently modified with part of the tumour dose being given by a rotation technique. Masses remaining after radiotherapy were kept under observation by further computed tomography of the abdomen. The maximal cross-sectional area of the retroperitoneal adenopathy was obtained from two measurements at right angles on scans obtained before and after chemotherapy, after radiotherapy and on scans obtained from subsequent follow-up.

### Results

Eighty-three patients had evidence of retroperitoneal adenopathy either at presentation or after a period of surveillance. Of these patients 47 had retroperitoneal lymphadenopathy which was less than 5 cm maximum transverse diameter on CT of the abdomen, 19 adenopathy more than 5 cm diameter and 17 had a palpable abdominal mass. Four patients were not assessed by CT at presentation but were clinically negative. Eighty-one patients received chemotherapy as follows: PVB 21, BeVIP 34, PEV 5 day 10, PEV 24 hour 14 and other combinations 2. Five patients received radiotherapy as a primary treatment and one mentally subnormal patient with advanced disease did not receive any treatment. The chemotherapy schedules used are shown in Table I. The outcome of treatment in the 87 patients is summarised in Table II. In 34 patients there was complete radiological resolution (CR) of the para-aortic disease and in 26 patients there was radiological evidence of a residual mass (RM) despite the return of tumour marker levels to normal. These patients were then considered for radiotherapy. In 21 patients there was progression of the para-aortic or other metastatic disease judged by persistent elevation of marker levels or radiological progression. In general these were patients with advanced large volume disease and all have subsequently progressed and died. There were two intercurrent and one treatment related deaths. Two patients did not have a reassessment of the para-aortic area after completion of treatment, one patient with para-aortic disease less than 5 cm at presentation is alive and disease-free, the other presented with advanced generalised disease including a palpable para-aortic mass. He was treated with chemotherapy followed by radiotherapy to a residual mediastinal mass but there was no re-

**Table I** Chemotherapy schedules

| | |
|---|---|
| PVB | |
| cis-platinum 20 mg m$^{-2}$ | days 1–5 |
| vinblastine 6 mg m$^{-2}$ | days 1 and 2 |
| bleomycin 15 mg b.d. | days 1–3 |
| BeVIP | |
| cis-platinum 20 mg m$^{-2}$ | days 1–5 |
| etoposide 100 mg m$^{-2}$ | days 1–3 |
| vinblastine 6 mg m$^{-2}$ | days 1 and 2 |
| bleomycin 30 mg i.m. | every 21 days |
| PEV | |
| cis-platinum 20 mg m$^{-2}$ | days 1–5 |
| etoposide 100 mg m$^{-2}$ | days 1–3 |
| vinblastine 10 mg | days 1 and 2 |
| PEV 24 h | |
| cis-platinum 100 mg m$^{-2}$ | 24 h infusion |
| etoposide 150 mg m$^{-2}$ | days 1 and 2 |
| vinblastine 10 mg | day 1 |

All cycles given every 3 weeks.

**Table II** Outcome of treatment in relation to size of initial para-aortic mass

| | | Outcome | | | | | |
|---|---|---|---|---|---|---|---|
| | | | | | Dead | | |
| *Initial mass* | *No.* | *CR* | *RM* | *CR not obtained* | *Inter-current* | *Treatment* | *Not rescanned* |
| <5 cm | 47 | 31 | 9 | 5 | 1 | 0 | 1 |
| >5 cm | 19 | 3 | 11 | 5 | 0 | 0 | 0 |
| Impalp | 4 | (1[a]) | 1 | 2 | 0 | 0 | 0 |
| Palp | 17 | 0 | 5 | 9 | 1 | 1 | 1 |
| Totals | 87 | 34 | 26 | 21 | 2 | 1 | 2 |

[a]Normal scan after treatment.
CR = complete radiological resolution; RM = radiological evidence of residual mass.

assessment of the para-aortic area. He subsequently had a generalised relapse including the para-aortic area.

### Complete response patients

The stages of the patients at treatment were IIA 17, IIB 1, III 1, IVA 6, IVB 7 and IVC 1. The sizes of the initial masses are shown in Table II. The pathological classifications are shown in Table III. The chemotherapy received by the CR patients was PVB 8, BEVIP 14, PEV 5 day 1, PEV 24 hour 7. Four patients received radiotherapy only as an initial treatment. Seven patients did not receive radiotherapy after chemotherapy and these all had minimal disease before treatment.

Twenty-eight of the 34 patients are alive with no recurrence. Three patients who had received radiotherapy to the para-aortic area only as an initial treatment relapsed in the lungs, two received successful chemotherapy and one died from treatment related septicaemia. One patient died from liver metastases five months after completing treatment, one patient died from cerebral relapse after three months and one patient who had received successful chemotherapy developed a tumour of the contralateral testis 38 months later and subsequently died from recurrent lung metastases. No patient had relapse in the para-aortic area. The results are summarised in Table IV.

**Table III** Pathological classification of patients having complete response (CR) of para-aortic disease and those leaving a residual mass (RM)

| | *CR* | *RM* |
|---|---|---|
| TD | 0 | 2 |
| MTI | 11 | 6 |
| MTU | 20 | 12 |
| MTT | 1 | 4 |
| YS | 1 | 1 |
| Unclass | 1 | 1 |
| Total | 34 | 26 |

TD = teratoma differentiated.
MTI = malignant teratoma intermediate.
MTU = malignant teratoma undifferentiated.
MTT = malignant teratoma trophoblastic.
YS = yolk sac tumour.

### Residual mass patients

The stages of the patients at treatment were IIA 6, IIB 5, III 3, IVA 3, IVB 6, IVC 3. The size of the initial para-aortic masses are shown in Table II. The pathological classifications of the patients are shown in Table III. The chemotherapy received by the RM patients was PVB 7, BEVIP 11, PEV 5 day

**Table IV** Outcome in relation to residual mass

| | *CR* | | *RM* | |
|---|---|---|---|---|
| *Total* | *34* | | *26* | |
| Radiotherapy | yes | no | yes | no |
| | 27 (4[b]) | 7 | 20 (2[a]) | 6 (2[a]) |
| Alive no recurrence | 21 (1[b]) | 7 | 16 | 4 |
| Relapsed (alive) | | | | |
| lung | 2[b] | | | |
| Relapsed (dead) | | | | |
| para-aortic | | | | 2[c] |
| stomach | | | 1 | |
| lung | 1[b] | | | |
| liver | 1 | | 1 | |
| brain | 1 | | 1 | |
| contralateral testis | 1 | | | |
| Dead (indeterminate) | | | 1 | |

[a]Laparotomy.
[b]XRT given as primary treatment.
[c]No XRT after chemotherapy.

4 and PEV 24 hour 4. Six patients did not receive radiotherapy following chemotherapy. One of these patients had signs of inferior vena caval obstruction and had a laparotomy immediately following chemotherapy but only fibrosis was found. The other five patients all had residual masses at other sites.

Of the twenty patients who had radiotherapy 16 are alive with no evidence of further recurrence, two of whom had a RPND with complete excision of the residual mass 6 and 9 months after completing radiotherapy because there was suspicion of enlargement of the persisting mass. Histology showed differentiated teratoma in one and necrotic material in the other. Three patients had relapsed, one in the stomach 34 months after completing treatment, the para-aortic area being negative at laparotomy, one in the liver with the residual mass unchanged after six months and one in the brain after 18 months. One patient died at home of a presumed myocardial infarction.

Of the six patients who did not have radiotherapy four are alive and well including one who had enlargement of the retroperitoneal mass and a supraclavicular node one year after completing chemotherapy. Biopsy of the node and the para-aortic mass showed differentiated teratoma but complete resection was not possible. Two patients had relapse in the para-aortic area judged by radiological evidence of relapse associated with rising tumour marker level 27 and 47 months after treatment. The cross-sectional areas of the para-aortic masses of those patients with a residual mass are shown in Table V before and after chemotherapy and at a recent assessment. Follow up scans were not available in 10 patients for the following reasons: RPND 3, died before assessment 2, too heavy for scanner 1, patient refused 2, relapsed in para-aortic area 2. Thus the fate of the para-aortic mass was followed in the remaining 16 patients. Seven had more than 75% reduction in the size of the mass during the period of observation and no change had taken place in 4 (Table VI).

**Table V** Cross-sectional areas of patients with residual masses

| *Cross-sectional areas of para-aortic masses (cm²)* | | | | |
|---|---|---|---|---|
| | | | *% Reduction* | |
| *Initial* | *p/CT* | *Recent* | *p/CT* | *Recent* |
| 314.2 | 141.1 | 102.1 | 55.0 | 27.8 |
| 181.4 | 44.2 | no scan | 75.6 | no scan |
| Palpable | 27.5 | 19.6 | — | (28.6)[a] |
| 62.8 | 19.6 | RPND | | RPND |
| 37.7 | 15.9 | 1.8 | 57.8 | 88.9 |
| 55.0 | 15.7 | RPND | 71.4 | RPND |
| 70.7 | 14.1 | 6.9 | 80.0 | 51.4 |
| 11.0 | 11.0 | 11.0 | 0 | 0(biopsy) |
| 8.2 | 8.2 | 8.2 | 0 | 0 |
| Palpable | 8.2 | 0.8 | — | 90.5 |
| Palpable | 7.1 | 3.1 | — | 55.6 |
| 47.1 | 7.1 | 3.1 | 85.0 | 55.6 |
| 75.4 | 4.9 | 0.0 | 93.5 | 99.4 |
| No scan | 4.9 | 33.0 | — | −572.0[a] |
| 15.7 | 4.9 | 1.6 | 68.7 | 68.0 |
| 19.6 | 3.1 | no scan | 84.0 | No scan |
| 19.6 | 3.1 | 3.1 | 84.0 | 0 |
| Palpable | 2.9 | 0.2 | — | 93.3 |
| 27.5 | 2.4 | 0.2 | 91.4 | 91.7 |
| 4.9 | 1.8 | 0.1 | 64.0 | 92.9 |
| Palpable | 1.8 | Died | — | Died |
| 12.6 | 1.8 | No scan | 85.9 | No scan |
| 7.1 | 1.8 | Died | 75.0 | Died |
| 4.7 | 1.6 | 0.2 | 66.7 | 87.5 |
| 1.6 | 1.6 | 1.6 | 0.0 | 0.0 |
| Palpable | | RPND | | RPND |

p/CT post-chemotherapy. RPND retroperitoneal node dissection.
[a]Relapsed.

**Table VI** % Reduction in cross-sectional areas of residual para-aortic masses from completion of chemotherapy to recent scan

| *% Reductions in residual masses* | |
|---|---|
| >75% | 7 |
| 50–75% | 4 |
| 26–50% | 1 |
| No change | 4 |
| Relapsed | 2 |

*Radiotherapy morbidity*

The morbidity of radiotherapy given as a primary treatment is well documented (Peckham *et al.*, 1981*b*). Only the 43 CR and RM patients receiving radiotherapy post-chemotherapy are considered here. The doses received are shown in Table VII. No patient failed to complete the planned radiotherapy treatment. During radiotherapy mild nausea, abdominal colic and diarrhoea were common but no patient developed symptoms severe enough to warrant interruption of treatment. Some myelosuppression during radiotherapy occurred in all patients. Four patients had grade 1 leuocopenia ($>2.0$ and $<3.0\times10^{9}\,l^{-1}$) and seven patients had grade 2 leucopenia ($>1.0$ and $<2.0\times10^{9}\,l^{-1}$). Two patients had brief interruption of treatments but there were no infective episodes. Five patients had thrombocytopenia between 50 and $100\times10^{9}\,l^{-1}$.

Subcutaneous fibrosis in the irradiated area developing within six months of the conclusion of radiotherapy was observed in 6 patients, in 3 of whom it was sufficiently severe to produce some restriction of movement. Fibrosis occurred in 4/8 patients in whom the subcutaneous dose exceeded 4,290 cGy in 20 fractions in 28 days and 2/5 patients with a subcutaneous dose greater than 3,750 cGy in 16 fractions over 21 days. In six patients treated in the latter part of the present series and in patients treated subsequently 50% of the dose to the para-aortic area has been delivered using a rotation technique and no further cases of subcutaneous fibrosis have been observed following this modification which has also significantly reduced myelosuppression.

**Table VII** Radiotherapy doses given post-chemotherapy

| *Midplane dose (cGy)* | *No. of fractions* | |
|---|---|---|
| | *16* | *20* |
| 4,000 | – | 33[a] |
| 3,750 | 2 | – |
| 3,500 | 3 | 4 |
| 3,000 | – | 1 |
| Total | 5 | 38 |

[a]50% of tumour dose given by rotation in 6 patients.

### Discussion

The aim of this study was to assess the results of radiotherapy to the retroperitoneum following chemotherapy in metastatic teratoma of the testis. The relapse rate in the retroperitoneum was very low – only 2/60 attaining CR or leaving a residual mass – neither of whom had received radiotherapy to the retroperitoneum. Although this was not a randomised study there was no retroperitoneal relapse in the 47 patients receiving radiotherapy post-chemotherapy whereas 2/11 not receiving radiotherapy or an immediate laparotomy relapsed. One further patient with a palpable para-aortic mass at presentation who did not have reassassment or radiotherapy of the para-aortic after chemotherapy subsequently relapsed in that area.

Many centres advocate a retroperitoneal node dissection (RPND) for residual masses following chemotherapy. These operations are tedious and may involve resection and or repair of the aorta, inferior vena cava, bowel or ureter (Blandy, 1985) since the object of surgery is complete removal of the residual mass, not merely a biopsy (Rowland & Donohoe, 1984). There may be considerable morbidity in the form of post-operative back pain and ejaculatory impotence. A complication rate of 25% and a mortality of 2–4% has been reported (Donohoe & Rowland, 1981; Skinner *et al.*, 1982). In patients receiving RPND following chemotherapy the incidence of active tumour his high. Rowland and Donohoe (1984) found tumour in 35% of patients following chemotherapy and Oliver *et al.* (1983) found that one third of cases had tumour. In a recent study (Vulgrin *et al.*, 1985) of patients treated by chemotherapy and RPND malignant elements were found in 10/25 patients achieving complete remission. In a review of the experience of several centres Brenner *et al.* (1982) found residual malignant elements in 32% of patients. Only 7% of patients with necrosis, fibrosis or benign teratoma relapsed compared with 61% having residual malignancy. Although Tait *et al.* (1984) suggest that the ultimate prognosis may relate to the completeness or otherwise of the resection, patients with active tumour still require further chemotherapy as surgery is not curative in its own right.

In the present study 50/60 patients survived more than two years with only two para-aortic recurrences in patients not receiving radiotherapy. This suggests that radiotherapy is effective in sterilising minimal residual active tumour following chemotherapy. Previous experience of treating stage II patients by radiotherapy supports this since relapse within the irradiated area of the abdomen was rare (Peckham *et al.*, 1977). The morbidity of a four week radical course of ratiotherapy is negligible in comparison with RPND (Peckham & Barrett, 1981*b*) and patients are spared further salvage chemotherapy. Although it may be argued that radiotherapy may compromise further chemotherapy the fields used in this study encompassed only the para-aortic area thereby keeping the volume of marrow irradiated to a minimum. This has been reduced further by delivering 50% of the tumour dose by a rotation technique rather than parallel opposed fields. This study also suggests that following radiotherapy residual masses may be safely observed since the majority will show a substantial reduction in size with the passage of time.

In conclusion this study suggests that the use of radiotherapy provides an alternative approach to the use of surgery post-chemotherapy in patients with para-aortic nodal disease. This approach spares patients the inconvenience and morbidity of a prolonged surgical procedure and the need for further chemotherapy should active tumour be found. Further studies are required to determine whether radiotherapy is required in all patients or only those in whom there is a residual mass.

### References

BLANDY, J.P. (1985). Testicular tumours: Role of surgery. *J. Royal Soc. Med.*, Suppl. No. 6, **78,** 32.

BRENNER, J., VUGRIN, D. & WHITMORE, W.F. (1982). Cytoreductive surgery for advanced non-seminomatous germ cell tumours of the testis. *Urology*, **19,** 571.

DONOHOE, J.P. & ROWLAND, R.G. (1981). Complications of retroperitoneal lymph node dissection. *J. Urol.*, **125,** 338.

EASSON, E.C. & POINTON, R.C.S. (1985). The treatment of malignant disease by radiotherapy. Appendix 2. *Clinical Staging*, p. 464. Springer-Verlag: Berlin.

GARNICK, M.B., CANNELLOS, G.P. & RICHIE, J.P. (1983). Treatment and surgical staging of testicular and primary extragonadal germ cell cancer. *JAMA.*, **250,** 1733.

GIBB, R. & READ, G. (1985). Radiotherapy of testicular tumours. In *The Treatment of Malignant Disease by Radiotherapy*, Easson, E.C. & Pointon, R.C.S. (eds) p. 341. Springer-Verlag: Berlin.

MUGGIA, F.M. (1985). Testicular cancer and the legacy of chemotherapy. *Cancer Chemother. Pharmacol.*, **15,** 1.

OLIVER, R.T.D., BLANDY, J.P., HENDRY, W.F., PRYOR, J.P., WILLIAMS, J.P. & HOPE-STONE, H.F. (1983). *Br. J. Urol.* **55,** 764.

PECKHAM, M.J., HENDRY, W.F., McELWAIN, T.J. & CALMAN, F.M.B. (1977). The multimodality treatment of testicular teratomas. In *Adjuvant Therapy of Cancer*, Salmon, S.E. & Jones, S.E. (eds) p. 305. North-Holland Publishing Company: Amsterdam, Oxford and New York.

PECKHAM, M.J. & BARRETT, A. (1981*a*). Radiotherapy in testicular teratoma. In *The Management of Testicular Tumours*, Peckham, M.J. (ed) *p*. 174. Edward Arnold: London.

PECKHAM, M.J. & BARRETT, A. (1981*b*). Radiotherapy in testicular teratoma. In *The Management of Testicular Tumours*, Peckham, M.J. (ed) p. 196. Edward Arnold: London.

PECKHAM, M.J. (1985). The management of testicular cancer. *Cancer Topics*, **5,** 66.

READ, G., JOHNSON, R.J., WILKINSON, P.M. & EDDLESTON, B. (1983). Prospective study of follow up alone in stage 1 teratoma of the testis. *Br. Med. J.*, **287,** 1503.

ROWLAND, R.G. & DONOHOE, J.P. (1984). *World J. Urol.* **2,** 48.

SKINNER, D.G., MELANUD, A. & LIESKOVSKY, G. (1982). Complications of thoraco-abdominal retroperitoneal lymph node dissection. *J. Urol.*, **127,** 1107.

TAIT, D., PECKHAM, M.J., HENDRY, W.F. & GOLDSHAW, P. (1984). Post-chemotherapy surgery in advanced non-seminomatous germ cell testicular tumours: The significance of histology with particular reference to differentiated (mature) teratoma. *Br. J. Cancer*, **50,** 601.

VULGRIN, D. & WHITMORE, W.F. (1985). The role of chemotherapy and surgery in the treatment of retroperitoneal metastases in advanced nonseminomatous testis cancer. *Cancer*, **55,** 1874.

WILKINSON, P.M. (1985). Chemotherapy for non-seminomatous germ cell tumours. *J. Royal Soc. Med.*, Suppl. No. 6, **78,** 43.